# Supplementary material for: Machine learning models predict the emergence of depression in Argentinean college students during periods of COVID-19 quarantine
Source: Front Psychiatry. 2024 Apr 16;15:1376784. doi: 10.3389/fpsyt.2024.1376784 (PMC11059062; doi:10.3389/fpsyt.2024.1376784)
Supplement: Supplementary file 1 [file DataSheet_1.docx]

**Supplementary files for “Machine Learning Models Predict the Emergence of Depression in Argentinean College Students during Periods of COVID-19 Quarantine”**

**INDEX**

| **Figure S1.** Description of the sampling design. | 3 |
| --- | --- |
| **Table S1.** Results of the hyperparameter tuning for the Linear Logistic Regression Classifier | 4 |
| **Figure S2.** Grid Search Results for the Linear Logistic Regression Classifier | 5 |
| **Table S2.** Results of the hyperparameter tuning for the Random Forest Classifier | 6 |
| **Table S3.** Results of the hyperparameter tuning for the Support Vector Machine Classifier | 10 |
| **Figure S3.** Grid Search Results for the Support Vector Machine Classifier | 12 |
| **Table S4.** Results of the hyperparameter tuning for the Ridge Regression | 13 |
| **Figure S4.** Grid Search Results for the Ridge Regression | 14 |
| **Table S5.** Results of the hyperparameter tuning for the Random Forest Regressor | 15 |
| **Figure S5.** Grid Search Results for the Support Vector Regressor | 19 |
| **Figure S6.** Confusion matrix plots comparing the performance of machine learning classifiers for depression | 20 |
| **Table S6.** Classification performance of machine learning algorithms and dummy baseline models on test set for predicting depression in college students | 21 |
| **Table S7.** Regression performance of machine learning algorithms and dummy baseline models on test set for predicting depression in college students | 22 |
| **Figure S7.** Correlation between actual and predicted values for each machine learning algorithm for the regression task on the test set | 23 |
| **Table S8.** Comparison of the predictive performance of multivariate (all features included) versus univariate (single feature) machine learning models in the classification task | 24 |
| **Table S9.** Comparison of the predictive performance of multivariate (all features included) versus univariate (single feature) machine learning models in the regression task | 25 |


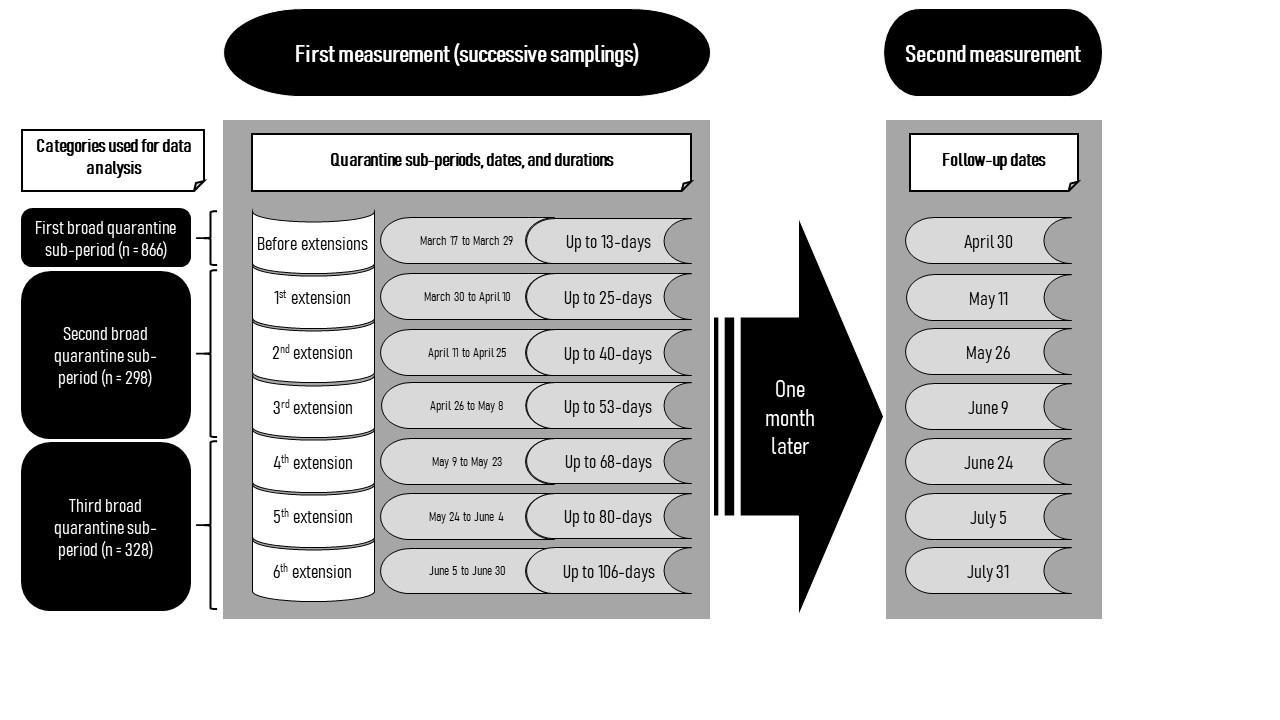


**Figure S1.** *Description of the sampling design*. The first measurement was carried out along successive samplings during the Argentinean quarantine sub-periods. These sub-periods were based on the dates of the Argentinean Government’s announcements on mandatory quarantine and their extensions. For the first measurement, successive samplings were carried out up to the sixth extension, corresponding to a quarantine of up to 106 days’ duration. The second measurement was carried out one month later. For data analysis, categories corresponding to three broad quarantine sub-periods were used.

| **Table S1.** Results of the hyperparameter tuning for the Linear Logistic Regression Classifier | | |
| --- | --- | --- |
|  | **Cross-validation mean validation score** | **Hyperparameters** |
| 0 | 0.795241 | C: 0.0001 |
| 1 | 0.797343 | C: 0.001 |
| 2 | 0.797071 | C: 0.01 |
| 3 | **0.798839** | **C: 0.1** |
| 4 | 0.798001 | C: 1 |
| 5 | 0.797562 | C: 10 |
| 6 | 0.797581 | C: 1000 |
| *Note*: The best hyperparameter selected for the linear logistic regression classifier is in bold. C: Regularization parameter. | | |

**
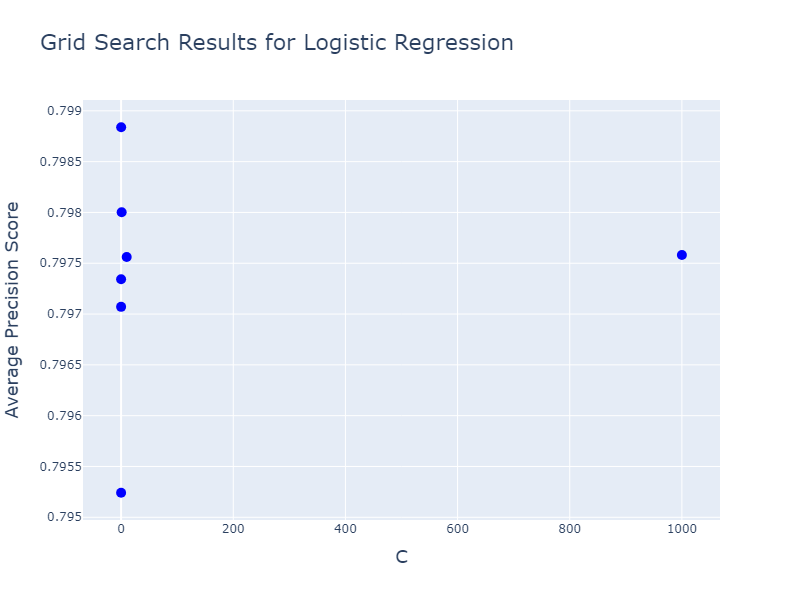
**

**Figure S2.** *Grid Search Results for the Linear Logistic Regression Classifier*. Scatter plot of the average precision scores for a linear logistic regression model during a grid search. The x-axis represents the values of C used in the hyperparameter search, and the y-axis represents the average precision scores obtained through cross-validation. *Notes*: C: Regularization parameter.

| **Table S2.** Results of the hyperparameter tuning for the Random Forest Classifier | |
| --- | --- |
| **Cross-validation mean validation score** | **Hyperparameters** |
| 0 0.783682  1 0.785611  2 0.788070  3 0.788372  4 0.785616  5 0.787058  6 0.788786  7 0.787448  8 0.777467  9 0.782851  10 0.785174  11 0.785656  12 0.784427  13 0.786116  14 0.788159  15 0.787215  16 0.785382  17 **0.789672**  18 0.786580  19 0.786613  20 0.779936  21 0.783464  22 0.784309  23 0.786428  24 0.782151  25 0.786537  26 0.782827  27 0.784599  28 0.782151  29 0.786537  30 0.782827  31 0.784599  32 0.782156  33 0.783767  34 0.783419  35 0.785211  36 0.767091  37 0.767097  38 0.764235  39 0.761589  40 0.765576  41 0.773114  42 0.768369  43 0.765794  44 0.763010  45 0.766327  46 0.772433  47 0.770786  48 0.755686  49 0.768513  50 0.767942  51 0.769764  52 0.768666  53 0.772143  54 0.771656  55 0.770101  56 0.763623  57 0.768942  58 0.768740  59 0.771337  60 0.765248  61 0.770760  62 0.773167  63 0.773620  64 0.765248  65 0.770760  66 0.773167  67 0.773620  68 0.769197  69 0.767412  70 0.772726  71 0.774051  72 0.744504  73 0.746366  74 0.750737  75 0.751043  76 0.763085  77 0.760036  78 0.757595  79 0.756354  80 0.757827  81 0.762440  82 0.764487  83 0.761871  84 0.755433  85 0.756757  86 0.761977  87 0.761748  88 0.765137  89 0.771540  90 0.767236  91 0.763770  92 0.760655  93 0.763965  94 0.765560  95 0.764605  96 0.764149  97 0.768032  98 0.768443  99 0.767748  100 0.764149  101 0.768032  102 0.768443  103 0.767748  104 0.758691  105 0.761830  106 0.769669  107 0.770717 | 'max_depth': 5, 'min_samples_leaf': 1, 'min_samples_split': 2, 'n_estimators': 50  'max_depth': 5, 'min_samples_leaf': 1, 'min_samples_split': 2, 'n_estimators': 100  'max_depth': 5, 'min_samples_leaf': 1, 'min_samples_split': 2, 'n_estimators': 500  'max_depth': 5, 'min_samples_leaf': 1, 'min_samples_split': 2, 'n_estimators': 1000  'max_depth': 5, 'min_samples_leaf': 1, 'min_samples_split': 5, 'n_estimators': 50  'max_depth': 5, 'min_samples_leaf': 1, 'min_samples_split': 5, 'n_estimators': 100  'max_depth': 5, 'min_samples_leaf': 1, 'min_samples_split': 5, 'n_estimators': 500  'max_depth': 5, 'min_samples_leaf': 1, 'min_samples_split': 5, 'n_estimators': 1000  'max_depth': 5, 'min_samples_leaf': 1, 'min_samples_split': 8, 'n_estimators': 50  'max_depth': 5, 'min_samples_leaf': 1, 'min_samples_split': 8, 'n_estimators': 100  'max_depth': 5, 'min_samples_leaf': 1, 'min_samples_split': 8, 'n_estimators': 500  'max_depth': 5, 'min_samples_leaf': 1, 'min_samples_split': 8, 'n_estimators': 1000  'max_depth': 5, 'min_samples_leaf': 2, 'min_samples_split': 2, 'n_estimators': 50  'max_depth': 5, 'min_samples_leaf': 2, 'min_samples_split': 2, 'n_estimators': 100  'max_depth': 5, 'min_samples_leaf': 2, 'min_samples_split': 2, 'n_estimators': 500  'max_depth': 5, 'min_samples_leaf': 2, 'min_samples_split': 2, 'n_estimators': 1000  'max_depth': 5, 'min_samples_leaf': 2, 'min_samples_split': 5, 'n_estimators': 50  **'max_depth': 5, 'min_samples_leaf': 2, 'min_samples_split': 5, 'n_estimators': 100**  'max_depth': 5, 'min_samples_leaf': 2, 'min_samples_split': 5, 'n_estimators': 500  'max_depth': 5, 'min_samples_leaf': 2, 'min_samples_split': 5, 'n_estimators': 1000  'max_depth': 5, 'min_samples_leaf': 2, 'min_samples_split': 8, 'n_estimators': 50  'max_depth': 5, 'min_samples_leaf': 2, 'min_samples_split': 8, 'n_estimators': 100  'max_depth': 5, 'min_samples_leaf': 2, 'min_samples_split': 8, 'n_estimators': 500  'max_depth': 5, 'min_samples_leaf': 2, 'min_samples_split': 8, 'n_estimators': 1000  'max_depth': 5, 'min_samples_leaf': 3, 'min_samples_split': 2, 'n_estimators': 50  'max_depth': 5, 'min_samples_leaf': 3, 'min_samples_split': 2, 'n_estimators': 100  'max_depth': 5, 'min_samples_leaf': 3, 'min_samples_split': 2, 'n_estimators': 500  'max_depth': 5, 'min_samples_leaf': 3, 'min_samples_split': 2, 'n_estimators': 1000  'max_depth': 5, 'min_samples_leaf': 3, 'min_samples_split': 5, 'n_estimators': 50  'max_depth': 5, 'min_samples_leaf': 3, 'min_samples_split': 5, 'n_estimators': 100  'max_depth': 5, 'min_samples_leaf': 3, 'min_samples_split': 5, 'n_estimators': 500  'max_depth': 5, 'min_samples_leaf': 3, 'min_samples_split': 5, 'n_estimators': 1000  'max_depth': 5, 'min_samples_leaf': 3, 'min_samples_split': 8, 'n_estimators': 50  'max_depth': 5, 'min_samples_leaf': 3, 'min_samples_split': 8, 'n_estimators': 100  'max_depth': 5, 'min_samples_leaf': 3, 'min_samples_split': 8, 'n_estimators': 500  'max_depth': 5, 'min_samples_leaf': 3, 'min_samples_split': 8, 'n_estimators': 1000  'max_depth': 10, 'min_samples_leaf': 1, 'min_samples_split': 2, 'n_estimators': 50  'max_depth': 10, 'min_samples_leaf': 1, 'min_samples_split': 2, 'n_estimators': 100  'max_depth': 10, 'min_samples_leaf': 1, 'min_samples_split': 2, 'n_estimators': 500  'max_depth': 10, 'min_samples_leaf': 1, 'min_samples_split': 2, 'n_estimators': 1000}  'max_depth': 10, 'min_samples_leaf': 1, 'min_samples_split': 5, 'n_estimators': 50  'max_depth': 10, 'min_samples_leaf': 1, 'min_samples_split': 5, 'n_estimators': 100  'max_depth': 10, 'min_samples_leaf': 1, 'min_samples_split': 5, 'n_estimators': 500  'max_depth': 10, 'min_samples_leaf': 1, 'min_samples_split': 5, 'n_estimators': 1000  'max_depth': 10, 'min_samples_leaf': 1, 'min_samples_split': 8, 'n_estimators': 50  'max_depth': 10, 'min_samples_leaf': 1, 'min_samples_split': 8, 'n_estimators': 100  'max_depth': 10, 'min_samples_leaf': 1, 'min_samples_split': 8, 'n_estimators': 500  'max_depth': 10, 'min_samples_leaf': 1, 'min_samples_split': 8, 'n_estimators': 1000  'max_depth': 10, 'min_samples_leaf': 2, 'min_samples_split': 2, 'n_estimators': 50  'max_depth': 10, 'min_samples_leaf': 2, 'min_samples_split': 2, 'n_estimators': 100  'max_depth': 10, 'min_samples_leaf': 2, 'min_samples_split': 2, 'n_estimators': 500  'max_depth': 10, 'min_samples_leaf': 2, 'min_samples_split': 2, 'n_estimators': 1000  'max_depth': 10, 'min_samples_leaf': 2, 'min_samples_split': 5, 'n_estimators': 50  'max_depth': 10, 'min_samples_leaf': 2, 'min_samples_split': 5, 'n_estimators': 100  'max_depth': 10, 'min_samples_leaf': 2, 'min_samples_split': 5, 'n_estimators': 500  'max_depth': 10, 'min_samples_leaf': 2, 'min_samples_split': 5, 'n_estimators': 1000  'max_depth': 10, 'min_samples_leaf': 2, 'min_samples_split': 8, 'n_estimators': 50  'max_depth': 10, 'min_samples_leaf': 2, 'min_samples_split': 8, 'n_estimators': 100  'max_depth': 10, 'min_samples_leaf': 2, 'min_samples_split': 8, 'n_estimators': 500  'max_depth': 10, 'min_samples_leaf': 2, 'min_samples_split': 8, 'n_estimators': 1000  'max_depth': 10, 'min_samples_leaf': 3, 'min_samples_split': 2, 'n_estimators': 50  'max_depth': 10, 'min_samples_leaf': 3, 'min_samples_split': 2, 'n_estimators': 100  'max_depth': 10, 'min_samples_leaf': 3, 'min_samples_split': 2, 'n_estimators': 500  'max_depth': 10, 'min_samples_leaf': 3, 'min_samples_split': 2, 'n_estimators': 1000  'max_depth': 10, 'min_samples_leaf': 3, 'min_samples_split': 5, 'n_estimators': 50  'max_depth': 10, 'min_samples_leaf': 3, 'min_samples_split': 5, 'n_estimators': 100  'max_depth': 10, 'min_samples_leaf': 3, 'min_samples_split': 5, 'n_estimators': 500  'max_depth': 10, 'min_samples_leaf': 3, 'min_samples_split': 5, 'n_estimators': 1000  'max_depth': 10, 'min_samples_leaf': 3, 'min_samples_split': 8, 'n_estimators': 50  'max_depth': 10, 'min_samples_leaf': 3, 'min_samples_split': 8, 'n_estimators': 100  'max_depth': 10, 'min_samples_leaf': 3, 'min_samples_split': 8, 'n_estimators': 500  'max_depth': 10, 'min_samples_leaf': 3, 'min_samples_split': 8, 'n_estimators': 1000  'max_depth': 15, 'min_samples_leaf': 1, 'min_samples_split': 2, 'n_estimators': 50  'max_depth': 15, 'min_samples_leaf': 1, 'min_samples_split': 2, 'n_estimators': 100  'max_depth': 15, 'min_samples_leaf': 1, 'min_samples_split': 2, 'n_estimators': 500  'max_depth': 15, 'min_samples_leaf': 1, 'min_samples_split': 2, 'n_estimators': 1000  'max_depth': 15, 'min_samples_leaf': 1, 'min_samples_split': 5, 'n_estimators': 50  'max_depth': 15, 'min_samples_leaf': 1, 'min_samples_split': 5, 'n_estimators': 100  'max_depth': 15, 'min_samples_leaf': 1, 'min_samples_split': 5, 'n_estimators': 500  'max_depth': 15, 'min_samples_leaf': 1, 'min_samples_split': 5, 'n_estimators': 1000  'max_depth': 15, 'min_samples_leaf': 1, 'min_samples_split': 8, 'n_estimators': 50  'max_depth': 15, 'min_samples_leaf': 1, 'min_samples_split': 8, 'n_estimators': 100  'max_depth': 15, 'min_samples_leaf': 1, 'min_samples_split': 8, 'n_estimators': 500  'max_depth': 15, 'min_samples_leaf': 1, 'min_samples_split': 8, 'n_estimators': 1000  'max_depth': 15, 'min_samples_leaf': 2, 'min_samples_split': 2, 'n_estimators': 50  'max_depth': 15, 'min_samples_leaf': 2, 'min_samples_split': 2, 'n_estimators': 100  'max_depth': 15, 'min_samples_leaf': 2, 'min_samples_split': 2, 'n_estimators': 500  'max_depth': 15, 'min_samples_leaf': 2, 'min_samples_split': 2, 'n_estimators': 1000  'max_depth': 15, 'min_samples_leaf': 2, 'min_samples_split': 5, 'n_estimators': 50  'max_depth': 15, 'min_samples_leaf': 2, 'min_samples_split': 5, 'n_estimators': 100  'max_depth': 15, 'min_samples_leaf': 2, 'min_samples_split': 5, 'n_estimators': 500  'max_depth': 15, 'min_samples_leaf': 2, 'min_samples_split': 5, 'n_estimators': 1000  'max_depth': 15, 'min_samples_leaf': 2, 'min_samples_split': 8, 'n_estimators': 50  'max_depth': 15, 'min_samples_leaf': 2, 'min_samples_split': 8, 'n_estimators': 100  'max_depth': 15, 'min_samples_leaf': 2, 'min_samples_split': 8, 'n_estimators': 500  'max_depth': 15, 'min_samples_leaf': 2, 'min_samples_split': 8, 'n_estimators': 1000  'max_depth': 15, 'min_samples_leaf': 3, 'min_samples_split': 2, 'n_estimators': 50  'max_depth': 15, 'min_samples_leaf': 3, 'min_samples_split': 2, 'n_estimators': 100  'max_depth': 15, 'min_samples_leaf': 3, 'min_samples_split': 2, 'n_estimators': 500  'max_depth': 15, 'min_samples_leaf': 3, 'min_samples_split': 2, 'n_estimators': 1000  'max_depth': 15, 'min_samples_leaf': 3, 'min_samples_split': 5, 'n_estimators': 50  'max_depth': 15, 'min_samples_leaf': 3, 'min_samples_split': 5, 'n_estimators': 100  'max_depth': 15, 'min_samples_leaf': 3, 'min_samples_split': 5, 'n_estimators': 500  'max_depth': 15, 'min_samples_leaf': 3, 'min_samples_split': 5, 'n_estimators': 1000  'max_depth': 15, 'min_samples_leaf': 3, 'min_samples_split': 8, 'n_estimators': 50  'max_depth': 15, 'min_samples_leaf': 3, 'min_samples_split': 8, 'n_estimators': 100  'max_depth': 15, 'min_samples_leaf': 3, 'min_samples_split': 8, 'n_estimators': 500  'max_depth': 15, 'min_samples_leaf': 3, 'min_samples_split': 8, 'n_estimators': 1000 |
| *Note*: The best combination of hyperparameters selected for the random forest classifier is in bold. max_depth: The maximum depth of the tree. min_samples_leaf: The minimum number of samples required to be at a leaf node. min_samples_split: The minimum number of samples required to split an internal node. n_estimators: The number of trees in the forest. | |

| **Table S3.** Results of the hyperparameter tuning for the Support Vector Machine Classifier | | |
| --- | --- | --- |
|  | **Cross-validation mean validation score** | **Hyperparameters** |
| 0 | 0.794651 | C: 0.01, gamma: 1e-05, kernel: rbf |
| 1 | 0.794631 | C: 0.01,'gamma: 0.0001, kernel: rbf |
| 2 | 0.794857 | C: 0.01, gamma: 0.001, kernel: rbf |
| 3 | 0.794826 | C: 0.01, gamma: 0.01, kernel: rbf |
| 4 | 0.791338 | C: 0.01, gamma: 0.1, kernel: rbf |
| 5 | 0.741115 | C: 0.01, gamma: 1, kernel: rbf |
| 6 | 0.626249 | C: 0.01, gamma: 10, kernel: rbf |
| 7 | 0.794651 | C: 0.1, gamma: 1e-05, kernel: rbf |
| 8 | 0.794631 | C: 0.1, gamma: 0.0001, kernel: rbf |
| 9 | 0.794725 | C: 0.1, gamma: 0.001, kernel: rbf |
| 10 | 0.796703 | C: 0.1, gamma: 0.01, kernel: rbf |
| 11 | 0.790996 | C: 0.1, gamma: 0.1, kernel: rbf |
| 12 | 0.744310 | C: 0.1, gamma: 1, kernel: rbf |
| 13 | 0.626249 | C: 0.1, gamma: 10, kernel: rbf |
| 14 | 0.794651 | C: 1, gamma: 1e-05, kernel: rbf |
| 15 | 0.794469 | C: 1, gamma: 0.0001, kernel: rbf |
| 16 | 0.796638 | C: 1, gamma: 0.001, kernel: rbf |
| 17 | 0.798668 | C: 1, gamma: 0.01, kernel: rbf |
| 18 | 0.783474 | C: 1, gamma: 0.1, kernel: rbf |
| 19 | 0.697051 | C: 1, gamma: 1, kernel: rbf |
| 20 | 0.605214 | C: 1, gamma: 10, kernel: rbf |
| 21 | 0.794469 | C: 10, gamma: 1e-05, kernel: rbf |
| 22 | 0.795930 | C: 10, gamma: 0.0001, kernel: rbf |
| 23 | 0.800951 | C: 10, gamma: 0.001, kernel: rbf |
| 24 | 0.792662 | C: 10, gamma: 0.01, kernel: rbf |
| 25 | 0.772164 | C: 10, gamma: 0.1, kernel: rbf |
| 26 | 0.624238 | C: 10, gamma: 1, kernel: rbf |
| 27 | 0.564755 | C: 10, gamma: 10, kernel: rbf |
| 28 | 0.795795 | C: 100, gamma: 1e-05, kernel: rbf |
| 29 | 0.801177 | C: 100, gamma: 0.0001, kernel: rbf |
| 30 | 0.795544 | C: 100, gamma: 0.001, kernel: rbf |
| 31 | 0.783134 | C: 100, gamma: 0.01, kernel: rbf |
| 32 | 0.726780 | C: 100, gamma: 0.1, kernel: rbf |
| 33 | 0.579707 | C: 100, gamma: 1, kernel: rbf |
| 34 | 0.563953 | C: 100, gamma: 10, kernel: rbf |
| 35 | 0.800709 | C: 500, gamma: 1e-05, kernel: rbf |
| 36 | 0.797627 | C: 500, gamma: 0.0001, kernel: rbf |
| 37 | 0.791916 | C: 500, gamma: 0.001, kernel: rbf |
| 38 | 0.778532 | C: 500, gamma: 0.01, kernel: rbf |
| 39 | 0.683220 | C: 500, gamma: 0.1, kernel: rbf |
| 40 | 0.572323 | C: 500, gamma: 1, kernel: rbf |
| 41 | 0.563153 | C: 500, gamma: 10, kernel: rbf |
| 42 | **0.801209** | **C: 1000, gamma: 1e-05, kernel: rbf** |
| 43 | 0.797197 | C: 1000, gamma: 0.0001, kernel: rbf |
| 44 | 0.790851 | C: 1000, gamma: 0.001, kernel: rbf |
| 45 | 0.777073 | C: 1000, gamma: 0.01, kernel: rbf |
| 46 | 0.675762 | C: 1000, gamma: 0.1, kernel: rbf |
| 47 | 0.574759 | C: 1000, gamma: 1, kernel: rbf |
| 48 | 0.562591 | C: 1000, gamma: 10, kernel: rbf |
| 49 | 0.800731 | C: 0.01, kernel: linear |
| 50 | 0.797639 | C: 0.1, kernel: linear |
| 51 | 0.796875 | C: 1, kernel: linear |
| 52 | 0.796778 | C: 10, kernel: linear |
| 53 | 0.796884 | C: 100, kernel: linear |
| 54 | 0.797044 | C: 500, kernel: linear |
| 55 | 0.797214 | C: 1000, kernel: linear |
| *Note*: The best hyperparameter combination selected for the support vector machine classifier is in bold. C: Regularization parameter. rbf: Radial basis function. | | |


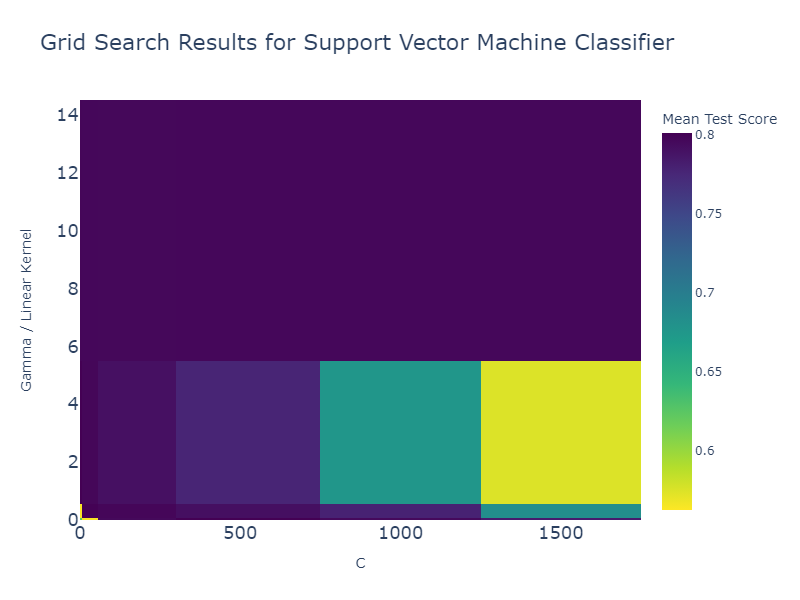


**Figure S3.** *Grid Search Results for the Support Vector Machine Classifier*. Heatmap of the performance of the support vector machine classifier across different hyperparameter values during a grid search. The x-axis represents the regularization parameter, C, while the y-axis corresponds to the kernel parameter, with values specified for the radial basis function (rbf) and linear kernels. The color intensity reflects the mean test score (average precision score), indicating the classifier’s average performance under different configurations. Warmer colors signify higher scores. *Notes*: C: Regularization parameter.

| **Table S4.** Results of the hyperparameter tuning for the Ridge Regression | | |
| --- | --- | --- |
|  | **Cross-validation mean validation score** | **Hyperparameters** |
| 0 | 0.513599 | alpha: 0.0001 |
| 1 | 0.513599 | alpha: 0.001 |
| 2 | 0.513600 | alpha: 0.01 |
| 3 | 0.513605 | alpha: 0.1 |
| 4 | 0.513658 | alpha: 1 |
| 5 | 0.514139 | alpha: 10 |
| 6 | **0.515707** | **alpha: 100** |
| 7 | 0.463237 | alpha: 1000 |
| *Note*: The best hyperparameter selected for the ridge regression is in bold. Alpha: Regularization parameter. | | |


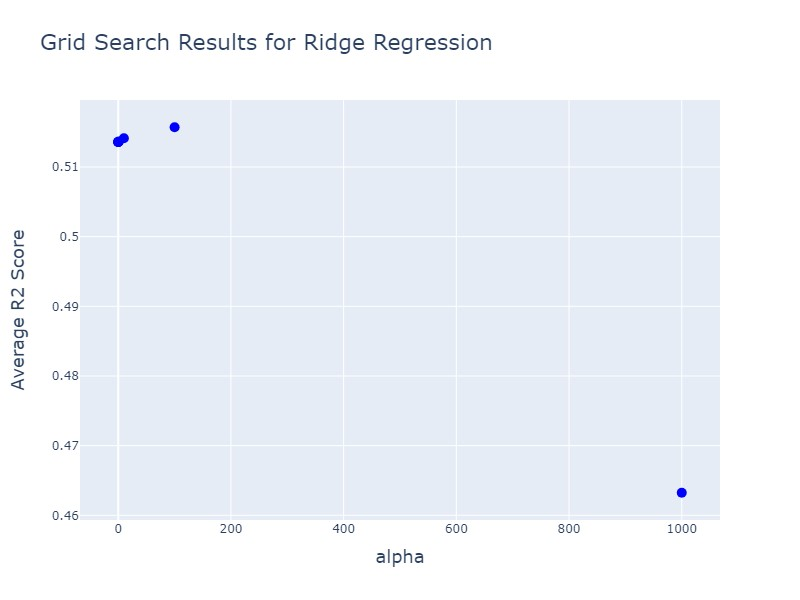


**Figure S4.** *Grid Search Results for the Ridge Regression*. Scatter plot of the average R2 scores for a ridge regression model during a grid search. The x-axis represents the values of the regularization parameter (alpha) used in the hyperparameter search, and the y-axis represents the average R2 scores obtained through cross-validation. *Notes*: Alpha: regularization parameter. R2: R-squared.

| **Table S5.** Results of the hyperparameter tuning for the Random Forest Regressor | |
| --- | --- |
| **Cross-validation mean validation score** | **Hyperparameters** |
| 0 0.490440  1 0.490503  2 0.490880  3 0.492089  4 0.493574  5 0.492713  6 0.492752  7 0.493907  8 0.494499  9 0.494350  10 0.494833  11 0.495730  12 0.491634  13 0.490389  14 0.489265  15 0.489948  16 0.494168  17 0.492618  18 0.491075  19 0.491378  20 0.495895  21 0.494897  22 0.493814  23 0.493930  24 0.495194  25 0.494826  26 0.493699  27 0.493373  28 0.495194  29 0.494826  30 0.493699  31 0.493373  32 **0.495977**  33 0.495509  34 0.494082  35 0.493627  36 0.461305  37 0.460461  38 0.462779  39 0.462137  40 0.470365  41 0.469628  42 0.469194  43 0.468311  44 0.471370  45 0.474069  46 0.473928  47 0.473386  48 0.459598  49 0.464173  50 0.464913  51 0.465073  52 0.464440  53 0.468230  54 0.468132  55 0.467757  56 0.469528  57 0.474359  58 0.473723  59 0.473475  60 0.467711  61 0.471557  62 0.472562  63 0.472082  64 0.467711  65 0.471557  66 0.472562  67 0.472082  68 0.470600  69 0.473985  70 0.474107  71 0.473583  72 0.446593  73 0.445005  74 0.446903  75 0.447014  76 0.456440  77 0.456503  78 0.456582  79 0.456450  80 0.462102  81 0.464549  82 0.464565  83 0.464583  84 0.447736  85 0.451813  86 0.454263  87 0.454400  88 0.452384  89 0.456571  90 0.458278  91 0.458122  92 0.460705  93 0.467032  94 0.466765  95 0.466650  96 0.460105  97 0.463155  98 0.465583  99 0.465346  100 0.460105  101 0.463155  102 0.465583  103 0.465346  104 0.464396  105 0.466965  106 0.468454  107 0.468100 | 'max_depth': 5, 'min_samples_leaf': 1, 'min_samples_split': 2, 'n_estimators': 50  'max_depth': 5, 'min_samples_leaf': 1, 'min_samples_split': 2, 'n_estimators': 100  'max_depth': 5, 'min_samples_leaf': 1, 'min_samples_split': 2, 'n_estimators': 500  'max_depth': 5, 'min_samples_leaf': 1, 'min_samples_split': 2, 'n_estimators': 1000  'max_depth': 5, 'min_samples_leaf': 1, 'min_samples_split': 5, 'n_estimators': 50  'max_depth': 5, 'min_samples_leaf': 1, 'min_samples_split': 5, 'n_estimators': 100  'max_depth': 5, 'min_samples_leaf': 1, 'min_samples_split': 5, 'n_estimators': 500  'max_depth': 5, 'min_samples_leaf': 1, 'min_samples_split': 5, 'n_estimators': 1000  'max_depth': 5, 'min_samples_leaf': 1, 'min_samples_split': 8, 'n_estimators': 50  'max_depth': 5, 'min_samples_leaf': 1, 'min_samples_split': 8, 'n_estimators': 100  'max_depth': 5, 'min_samples_leaf': 1, 'min_samples_split': 8, 'n_estimators': 500  'max_depth': 5, 'min_samples_leaf': 1, 'min_samples_split': 8, 'n_estimators': 1000  'max_depth': 5, 'min_samples_leaf': 2, 'min_samples_split': 2, 'n_estimators': 50  'max_depth': 5, 'min_samples_leaf': 2, 'min_samples_split': 2, 'n_estimators': 100  'max_depth': 5, 'min_samples_leaf': 2, 'min_samples_split': 2, 'n_estimators': 500  'max_depth': 5, 'min_samples_leaf': 2, 'min_samples_split': 2, 'n_estimators': 1000  'max_depth': 5, 'min_samples_leaf': 2, 'min_samples_split': 5, 'n_estimators': 50  'max_depth': 5, 'min_samples_leaf': 2, 'min_samples_split': 5, 'n_estimators': 100  'max_depth': 5, 'min_samples_leaf': 2, 'min_samples_split': 5, 'n_estimators': 500  'max_depth': 5, 'min_samples_leaf': 2, 'min_samples_split': 5, 'n_estimators': 1000  'max_depth': 5, 'min_samples_leaf': 2, 'min_samples_split': 8, 'n_estimators': 50  'max_depth': 5, 'min_samples_leaf': 2, 'min_samples_split': 8, 'n_estimators': 100  'max_depth': 5, 'min_samples_leaf': 2, 'min_samples_split': 8, 'n_estimators': 500  'max_depth': 5, 'min_samples_leaf': 2, 'min_samples_split': 8, 'n_estimators': 1000  'max_depth': 5, 'min_samples_leaf': 3, 'min_samples_split': 2, 'n_estimators': 50  'max_depth': 5, 'min_samples_leaf': 3, 'min_samples_split': 2, 'n_estimators': 100  'max_depth': 5, 'min_samples_leaf': 3, 'min_samples_split': 2, 'n_estimators': 500  'max_depth': 5, 'min_samples_leaf': 3, 'min_samples_split': 2, 'n_estimators': 1000  'max_depth': 5, 'min_samples_leaf': 3, 'min_samples_split': 5, 'n_estimators': 50  'max_depth': 5, 'min_samples_leaf': 3, 'min_samples_split': 5, 'n_estimators': 100  'max_depth': 5, 'min_samples_leaf': 3, 'min_samples_split': 5, 'n_estimators': 500  'max_depth': 5, 'min_samples_leaf': 3, 'min_samples_split': 5, 'n_estimators': 1000  **'max_depth': 5, 'min_samples_leaf': 3, 'min_samples_split': 8, 'n_estimators': 50**  'max_depth': 5, 'min_samples_leaf': 3, 'min_samples_split': 8, 'n_estimators': 100  'max_depth': 5, 'min_samples_leaf': 3, 'min_samples_split': 8, 'n_estimators': 500  'max_depth': 5, 'min_samples_leaf': 3, 'min_samples_split': 8, 'n_estimators': 1000  'max_depth': 10, 'min_samples_leaf': 1, 'min_samples_split': 2, 'n_estimators': 50  'max_depth': 10, 'min_samples_leaf': 1, 'min_samples_split': 2, 'n_estimators': 100  'max_depth': 10, 'min_samples_leaf': 1, 'min_samples_split': 2, 'n_estimators': 500  'max_depth': 10, 'min_samples_leaf': 1, 'min_samples_split': 2, 'n_estimators': 1000  'max_depth': 10, 'min_samples_leaf': 1, 'min_samples_split': 5, 'n_estimators': 50  'max_depth': 10, 'min_samples_leaf': 1, 'min_samples_split': 5, 'n_estimators': 100  'max_depth': 10, 'min_samples_leaf': 1, 'min_samples_split': 5, 'n_estimators': 500  'max_depth': 10, 'min_samples_leaf': 1, 'min_samples_split': 5, 'n_estimators': 1000  'max_depth': 10, 'min_samples_leaf': 1, 'min_samples_split': 8, 'n_estimators': 50  'max_depth': 10, 'min_samples_leaf': 1, 'min_samples_split': 8, 'n_estimators': 100  'max_depth': 10, 'min_samples_leaf': 1, 'min_samples_split': 8, 'n_estimators': 500  'max_depth': 10, 'min_samples_leaf': 1, 'min_samples_split': 8, 'n_estimators': 1000  'max_depth': 10, 'min_samples_leaf': 2, 'min_samples_split': 2, 'n_estimators': 50  'max_depth': 10, 'min_samples_leaf': 2, 'min_samples_split': 2, 'n_estimators': 100  'max_depth': 10, 'min_samples_leaf': 2, 'min_samples_split': 2, 'n_estimators': 500  'max_depth': 10, 'min_samples_leaf': 2, 'min_samples_split': 2, 'n_estimators': 1000  'max_depth': 10, 'min_samples_leaf': 2, 'min_samples_split': 5, 'n_estimators': 50  'max_depth': 10, 'min_samples_leaf': 2, 'min_samples_split': 5, 'n_estimators': 100  'max_depth': 10, 'min_samples_leaf': 2, 'min_samples_split': 5, 'n_estimators': 500  'max_depth': 10, 'min_samples_leaf': 2, 'min_samples_split': 5, 'n_estimators': 1000}  'max_depth': 10, 'min_samples_leaf': 2, 'min_samples_split': 8, 'n_estimators': 50  'max_depth': 10, 'min_samples_leaf': 2, 'min_samples_split': 8, 'n_estimators': 100  'max_depth': 10, 'min_samples_leaf': 2, 'min_samples_split': 8, 'n_estimators': 500  'max_depth': 10, 'min_samples_leaf': 2, 'min_samples_split': 8, 'n_estimators': 1000  'max_depth': 10, 'min_samples_leaf': 3, 'min_samples_split': 2, 'n_estimators': 50  'max_depth': 10, 'min_samples_leaf': 3, 'min_samples_split': 2, 'n_estimators': 100  'max_depth': 10, 'min_samples_leaf': 3, 'min_samples_split': 2, 'n_estimators': 500  'max_depth': 10, 'min_samples_leaf': 3, 'min_samples_split': 2, 'n_estimators': 1000  'max_depth': 10, 'min_samples_leaf': 3, 'min_samples_split': 5, 'n_estimators': 50  'max_depth': 10, 'min_samples_leaf': 3, 'min_samples_split': 5, 'n_estimators': 100  'max_depth': 10, 'min_samples_leaf': 3, 'min_samples_split': 5, 'n_estimators': 500  'max_depth': 10, 'min_samples_leaf': 3, 'min_samples_split': 5, 'n_estimators': 1000  'max_depth': 10, 'min_samples_leaf': 3, 'min_samples_split': 8, 'n_estimators': 50  'max_depth': 10, 'min_samples_leaf': 3, 'min_samples_split': 8, 'n_estimators': 100  'max_depth': 10, 'min_samples_leaf': 3, 'min_samples_split': 8, 'n_estimators': 500  'max_depth': 10, 'min_samples_leaf': 3, 'min_samples_split': 8, 'n_estimators': 1000  'max_depth': 15, 'min_samples_leaf': 1, 'min_samples_split': 2, 'n_estimators': 50  'max_depth': 15, 'min_samples_leaf': 1, 'min_samples_split': 2, 'n_estimators': 100  'max_depth': 15, 'min_samples_leaf': 1, 'min_samples_split': 2, 'n_estimators': 500  'max_depth': 15, 'min_samples_leaf': 1, 'min_samples_split': 2, 'n_estimators': 1000  'max_depth': 15, 'min_samples_leaf': 1, 'min_samples_split': 5, 'n_estimators': 50  'max_depth': 15, 'min_samples_leaf': 1, 'min_samples_split': 5, 'n_estimators': 100  'max_depth': 15, 'min_samples_leaf': 1, 'min_samples_split': 5, 'n_estimators': 500  'max_depth': 15, 'min_samples_leaf': 1, 'min_samples_split': 5, 'n_estimators': 1000  'max_depth': 15, 'min_samples_leaf': 1, 'min_samples_split': 8, 'n_estimators': 50  'max_depth': 15, 'min_samples_leaf': 1, 'min_samples_split': 8, 'n_estimators': 100  'max_depth': 15, 'min_samples_leaf': 1, 'min_samples_split': 8, 'n_estimators': 500  'max_depth': 15, 'min_samples_leaf': 1, 'min_samples_split': 8, 'n_estimators': 1000  'max_depth': 15, 'min_samples_leaf': 2, 'min_samples_split': 2, 'n_estimators': 50  'max_depth': 15, 'min_samples_leaf': 2, 'min_samples_split': 2, 'n_estimators': 100  'max_depth': 15, 'min_samples_leaf': 2, 'min_samples_split': 2, 'n_estimators': 500  'max_depth': 15, 'min_samples_leaf': 2, 'min_samples_split': 2, 'n_estimators': 1000  'max_depth': 15, 'min_samples_leaf': 2, 'min_samples_split': 5, 'n_estimators': 50  'max_depth': 15, 'min_samples_leaf': 2, 'min_samples_split': 5, 'n_estimators': 100  'max_depth': 15, 'min_samples_leaf': 2, 'min_samples_split': 5, 'n_estimators': 500  'max_depth': 15, 'min_samples_leaf': 2, 'min_samples_split': 5, 'n_estimators': 1000  'max_depth': 15, 'min_samples_leaf': 2, 'min_samples_split': 8, 'n_estimators': 50  'max_depth': 15, 'min_samples_leaf': 2, 'min_samples_split': 8, 'n_estimators': 100  'max_depth': 15, 'min_samples_leaf': 2, 'min_samples_split': 8, 'n_estimators': 500  'max_depth': 15, 'min_samples_leaf': 2, 'min_samples_split': 8, 'n_estimators': 1000  'max_depth': 15, 'min_samples_leaf': 3, 'min_samples_split': 2, 'n_estimators': 50  'max_depth': 15, 'min_samples_leaf': 3, 'min_samples_split': 2, 'n_estimators': 100  'max_depth': 15, 'min_samples_leaf': 3, 'min_samples_split': 2, 'n_estimators': 500  'max_depth': 15, 'min_samples_leaf': 3, 'min_samples_split': 2, 'n_estimators': 1000  'max_depth': 15, 'min_samples_leaf': 3, 'min_samples_split': 5, 'n_estimators': 50  'max_depth': 15, 'min_samples_leaf': 3, 'min_samples_split': 5, 'n_estimators': 100  'max_depth': 15, 'min_samples_leaf': 3, 'min_samples_split': 5, 'n_estimators': 500  'max_depth': 15, 'min_samples_leaf': 3, 'min_samples_split': 5, 'n_estimators': 1000  'max_depth': 15, 'min_samples_leaf': 3, 'min_samples_split': 8, 'n_estimators': 50  'max_depth': 15, 'min_samples_leaf': 3, 'min_samples_split': 8, 'n_estimators': 100  'max_depth': 15, 'min_samples_leaf': 3, 'min_samples_split': 8, 'n_estimators': 500  'max_depth': 15, 'min_samples_leaf': 3, 'min_samples_split': 8, 'n_estimators': 1000 |
| *Notes*: The best combination of hyperparameters selected for the random forest regressor is in bold. max_depth: The maximum depth of the tree. min_samples_leaf: The minimum number of samples required to be at a leaf node. min_samples_split: The minimum number of samples required to split an internal node. n_estimators: The number of trees in the forest. | |


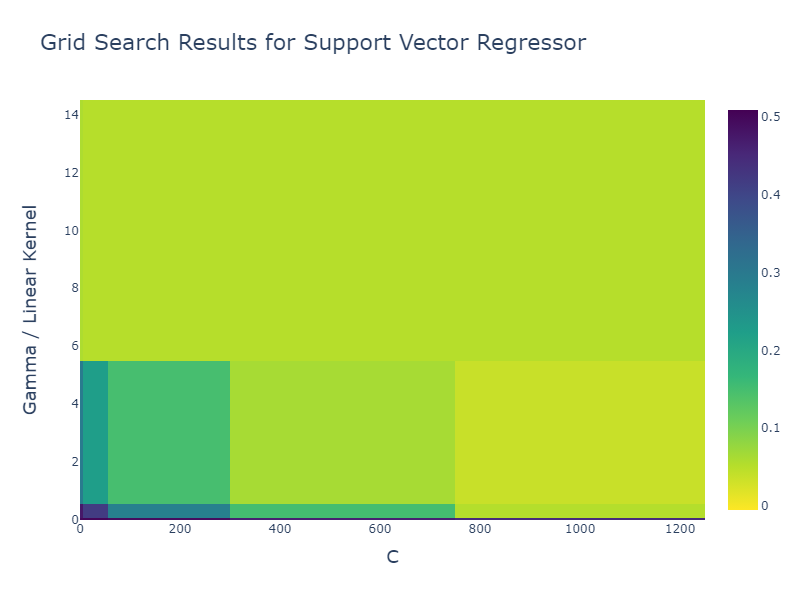


**Figure S5.** *Grid Search Results for the Support Vector Regressor (SVR)*. Heatmap of the performance of the support vector machine regressor across different hyperparameter values during a grid search. The x-axis represents the values of the regularization parameter, C. The y-axis displays the values of the hyperparameter Gamma for the radial basis function (rbf) kernel, or indicates linear for the linear kernel. The color of each cell corresponds to the mean R2 score achieved during cross-validation for the respective combination of C, Gamma, and kernel type. Darker colors represent higher mean test scores. *Notes*: C: Regularization parameter. R2: R-squared.


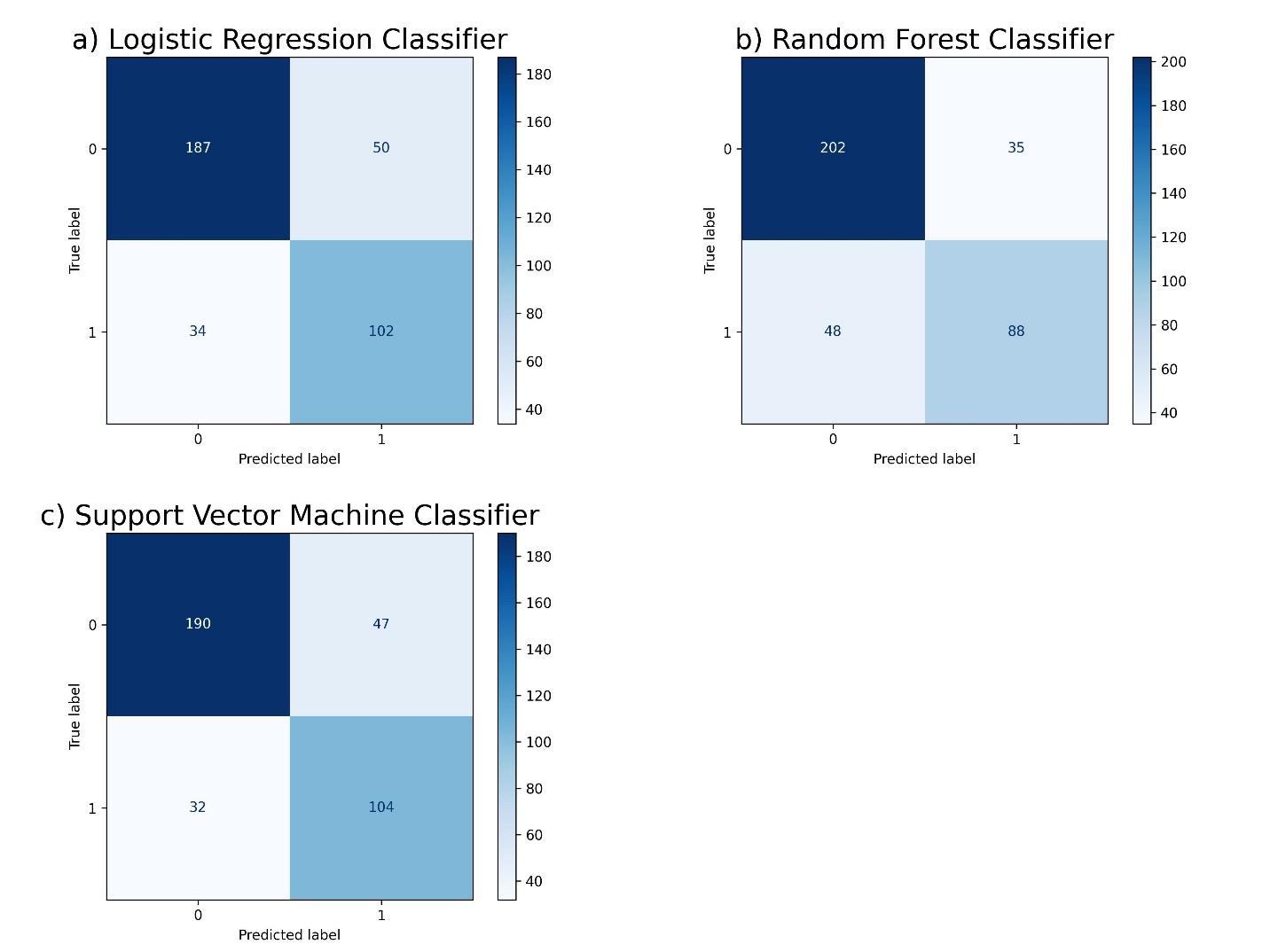


**Figure S6.** *Confusion matrix plots comparing the performance of machine learning classifiers for depression*. Each plot shows the number of correct and incorrect predictions of depression labels (1 = depressed, 0 = non-depressed). Darker color indicates a higher number of correct predictions. **a**) Linear Logistic Regression Classifier. **b**) Random Forest Classifier. **c**) Support Vector Machine Classifier.

| **Table S6.** Classification performance of machine learning algorithms and dummy baseline models on the test set for predicting depression in college students | | | | | | | |
| --- | --- | --- | --- | --- | --- | --- | --- |
| **Dummy (baselines) and classification models** | **Performance metrics for baseline and machine learning classification models on the test set** | | | | | | |
|  | **AUPRC** | **AUROC** | **Balanced accuracy** | **1 - Brier loss** | **F1 score** | **Precision** | **Recall** |
| Uniform Random Baseline | **0.36**  (0.33, 0.40) | **0.50**  (0.50, 0.50) | **0.50**  (0.45, 0.55) | **0.75**  (0.75, 0.75) | **0.43**  (0.37, 0.49) | **0.36**  (0.30, 0.43) | **0.52**  (0.46, 0.58) |
| Most Frequent Baseline | **0.36**  (0.33, 0.40) | **0.50**  (0.50, 0.50) | **0.50**  (0.50, 0.50) | **0.64**  (0.60, 0.67) | **0.00**  (0.00, 0.00) | **1.00**  (1.00, 1.00) | **0.00**  (0.00, 0.00) |
| Stratified Random Baseline | **0.36**  (0.32, 0.42) | **0.50**  (0.44, 0.55) | **0.50**  (0.44, 0.55) | **0.54**  (0.49, 0.58) | **0.36**  (0.30, 0.43) | **0.36**  (0.29, 0.45) | **0.36**  (0.29, 0.42) |
| Linear Logistic Regression | **0.76**  (0.69, 0.81) | **0.85**  (0.80, 0.88) | **0.77**  (0.72, 0.80) | **0.84**  (0.82, 0.86) | **0.71**  (0.65, 0.75) | **0.67**  (0.60, 0.73) | **0.75**  (0.67, 0.82) |
| Random Forest Classifier | **0.73**  (0.66, 0.80) | **0.83**  (0.79, 0.86) | **0.75**  (0.72, 0.79) | **0.84**  (0.82, 0.85) | **0.68**  (0.63, 0.72) | **0.72**  (0.65, 0.78) | **0.65**  (0.58, 0.72) |
| Support Vector Machine Classifier | **0.76**  (0.69, 0.81) | **0.85**  (0.80, 0.88) | **0.78**  (0.74, 0.82) | **0.85**  (0.83, 0.86) | **0.72**  (0.67, 0.76) | **0.69**  (0.63, 0.76) | **0.76**  (0.70, 0.83) |
| *Notes*: For the baselines and machine learning classification models in the test set, the mean scores obtained from model predictions across 100 simulated samplings using the bootstrap method are indicated in bold, with the lower and upper limits of 95% confidence interval in parentheses. The *1 - Brier loss* is presented as the inverse of the Brier score to ensure higher values consistently denote better performance across all methods. Original values of the Brier loss score are as follows: Uniform random baseline 0.25 (0.25, 0.25), Most frequent baseline 0.36 (0.33, 0.40), Stratified random baseline 0.46 (0.42, 0.51), Linear logistic regression 0.16 (0.14, 0.18), Random forest classifier 0.16 (0.15, 0.18), and SVM classifier 0.15 (0.14, 0.17). *Abbreviations*: AUPRC: Area Under the Precision-Recall Curve. AUROC: Area Under the Receiver Operating Characteristic Curve. | | | | | | | |

| **Table S7.** Regression performance of machine learning algorithms and dummy baseline models on the test set for predicting depression in college students | | | |
| --- | --- | --- | --- |
| **Dummy (baselines) and regression models** | **Performance metrics for baseline and machine learning regression models on the test set** | | |
|  | **R-squared** | **Mean Absolute Error** | **Mean Squared Error** |
| Randomly Shuffled Baseline | **-1.02**  (-1.33, -0.78) | **1.08**  (1.01, 1.17) | **1.85**  (1.65, 2.13) |
| Mean Baseline | **-0.003**  (-0.01, -0.00001) | **0.78**  (0.72, 0.85) | **0.92**  (0.77, 1.10) |
| Median Baseline | **-0.003**  (-0.01, -0.00002) | **0.78**  (0.72, 0.85) | **0.92**  (0.77, 1.10) |
| Ridge Regression | **0.56**  (0.45, 0.63) | **0.50**  (0.46, 0.54) | **0.40**  (0.34, 0.47) |
| Random Forest Regressor | **0.50**  (0.40, 0.59) | **0.52**  (0.48, 0.56) | **0.45**  (0.38, 0.55) |
| Support Vector Regressor | **0.56**  (0.45, 0.64) | **0.50**  (0.46, 0.53) | **0.40**  (0.33, 0.47) |
| *Notes*: For the baselines and machine learning regression models in the test set, the mean scores obtained from model predictions across 100 simulated samplings using the bootstrap method are indicated in bold, with the lower and upper limits of 95% confidence interval in parentheses. | | | |


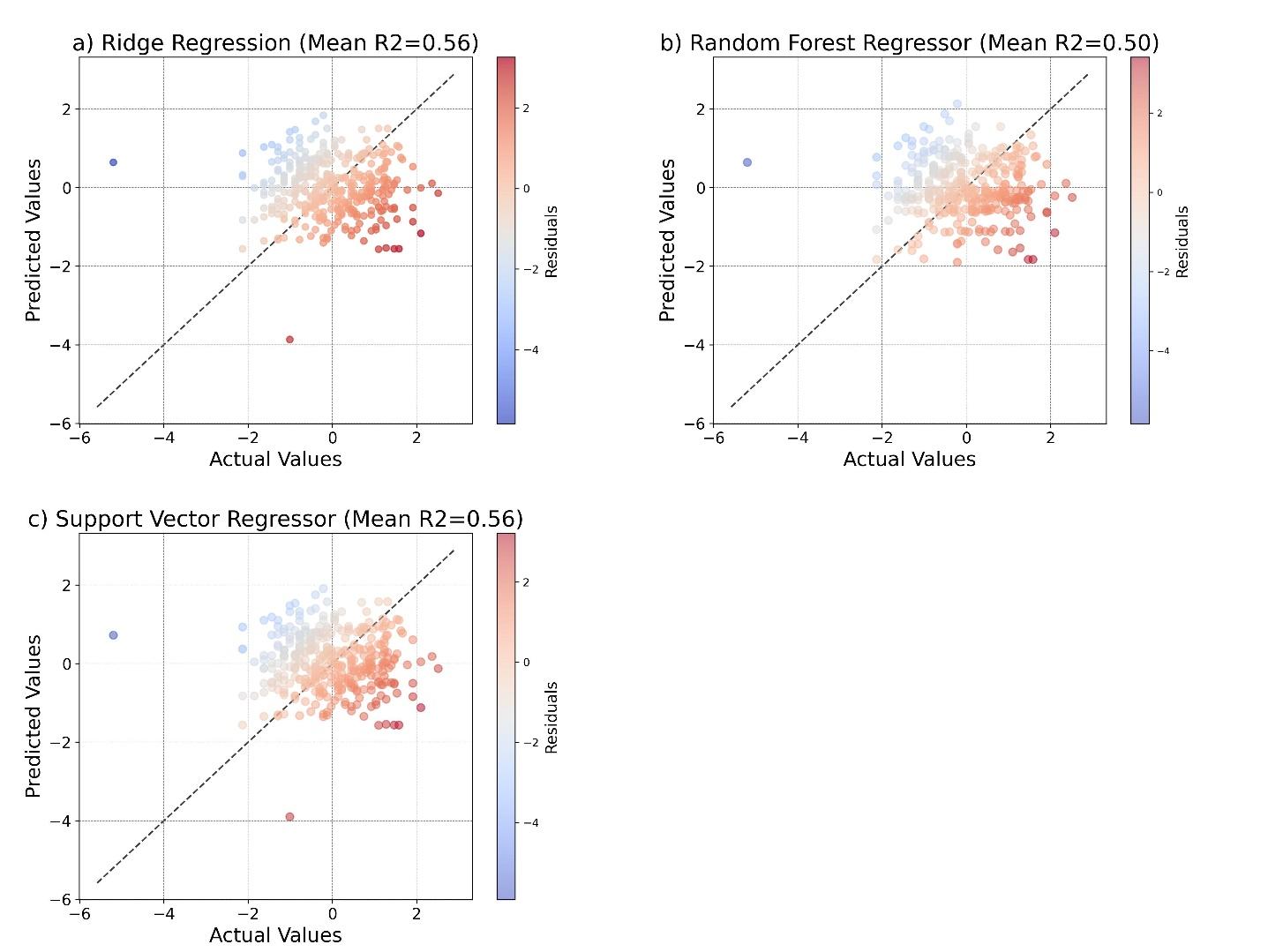


**Figure S7.** *Correlation between actual and predicted values for each machine learning algorithm for the regression task on the test set*. Plots show actual and predicted depression scores of three regression models for individual study participants alongside color-coded residual values. **a**) Ridge Regression. **b**) Random Forest Regressor. **c**) Support Vector Regressor.

| **Table S8.** Comparison of the predictive performance of multivariate (all features included) versus univariate (single feature) machine learning models in the classification task | | | | |
| --- | --- | --- | --- | --- |
|  |  | **Mean AUPRC scores (95% CI lower, upper)** | | |
|  | **Features** | **Linear logistic regression** | **Random forest classifier** | **Support vector machine classifier** |
| Multivariate model (includes all features) | | 0.76 (0.69, 0.81) | 0.73 (0.66, 0.80) | 0.76 (0.69, 0.81) |
| Univariate models | Depression T1 | 0.72 (0.64, 0.79) | 0.74 (0.68, 0.79) | 0.72 (0.64, 0.79) |
|  | Anxiety | 0.71 (0.64, 0.78) | 0.68 (0.60, 0.75) | 0.71 (0.64, 0.78) |
|  | Quarantine sub-period | 0.37 (0.33, 0.42) | 0.37 (0.33, 0.42) | 0.41 (0.35, 0.47) |
|  | Sex | 0.38 (0.34, 0.42) | 0.38 (0.34, 0.42) | 0.38 (0.34, 0.42) |
|  | Age | 0.43 (0.37, 0.50) | 0.41 (0.35, 0.48) | 0.43 (0.37, 0.50) |
|  | Mental disorder history | 0.46 (0.40, 0.52) | 0.46 (0.40, 0.52) | 0.46 (0.40, 0.52) |
|  | Suicidal behavior history | 0.46 (0.39, 0.52) | 0.46 (0.39, 0.52) | 0.46 (0.39, 0.52) |
| *Abbreviations*: AUPRC: Area Under the Precision-Recall Curve. CI: Confidence intervals. T1: First measurement or measurement at time one. | | | | |

| **Table S9.** Comparison of the predictive performance of multivariate (all features included) versus univariate (single feature) machine learning models in the regression task | | | | | |
| --- | --- | --- | --- | --- | --- |
|  | |  | **Mean R2 scores (95% CI lower, upper)** | | |
|  |  | **Features** | **Ridge regression** | **Random forest regressor** | **Support vector regressor** |
| Multivariate model (includes all features) | | | 0.56 (0.44, 0.64) | 0.50 (0.40, 0.59) | 0.56 (0.45, 0.64) |
| Univariate models | Depression T1 | | 0.48 (0.39, 0.57) | 0.46 (0.31, 0.58) | 0.33 (0.29, 0.38) |
|  | Anxiety | | 0.39 (0.30, 0.47) | 0.37 (0.25, 0.46) | 0.27 (0.22, 0.32) |
|  | Quarantine sub-period | | -0.00 (-0.02, 0.00) | -0.00 (-0.02, 0.00) | -0.04 (-0.08, -0.01) |
|  | Sex | | 0.01 (-0.02, 0.03) | 0.01 (-0.02, 0.03) | -0.03 (-0.08, -0.01) |
|  | Age | | 0.03 (0.00, 0.05) | 0.02 (-0.01, 0.04) | -0.01 (-0.05, 0.01) |
|  | Mental disorder history | | 0.03 (-0.02, 0.07) | 0.03 (-0.02, 0.07) | -0.01 (-0.06, 0.01) |
|  | Suicidal behavior history | | 0.13 (0.07, 0.20) | 0.13 (0.07, 0.20) | 0.02 (-0.02, 0.05) |
| *Abbreviations*: R2: R-squared. CI: Confidence intervals. T1: First measurement or measurement at time one. | | | | | |
